# Identification of Hub Biomarkers and Immune-Related Pathways Participating in the Progression of Antineutrophil Cytoplasmic Antibody-Associated Glomerulonephritis

**DOI:** 10.3389/fimmu.2021.809325

**Published:** 2022-01-05

**Authors:** Meng-Di Xia, Rui-Ran Yu, Dong-Ming Chen

**Affiliations:** ^1^ Department of Nephrology, The Second Clinical Medical Institution of North Sichuan Medical College (Nanchong Central Hospital) and Nanchong Key Laboratory of Basic Science & Clinical Research on Chronic Kidney Disease, Nanchong, China; ^2^ Department of Nephrology and Medical Intensive Care, Charité – Universtitätsmedizin Berlin, Cooperate Member of Freie Universität and Humboldt Universität, Hindenburgdamm, Berlin, Germany; ^3^ Department of Oncology, Anqing First People’s Hospital of Anhui Medical University, Anqing, China; ^4^ Department of Neurosurgery, The First Affiliated Hospital of Anhui University of Traditional Chinese Medicine, Hefei, China; ^5^ Charité – Universtitätsmedizin Berlin, Cooperate Member of Freie Universität and Humboldt Universität, Berlin, Germany

**Keywords:** antineutrophil cytoplasmic antibody-associated vasculitis, biomarker, immune cell infiltration, LASSO, weighted gene co-expression network analysis, immune-related pathways

## Abstract

**Background:**

Antineutrophil cytoplasmic antibody (ANCA)-associated vasculitis (AAV) is a systemic autoimmune disease that generally induces the progression of rapidly progressive glomerulonephritis (GN). The purpose of this study was to identify key biomarkers and immune-related pathways involved in the progression of ANCA-associated GN (ANCA-GN) and their relationship with immune cell infiltration.

**Methods:**

Gene microarray data were downloaded from the Gene Expression Omnibus (GEO). Hub markers for ANCA-GN were mined based on differential expression analysis, weighted gene co-expression network analysis (WGCNA) and lasso regression, followed by Gene Ontology (GO), Kyoto Encyclopedia of Genes and Genomes (KEGG) and Gene Set Enrichment Analysis (GSEA) of the differential genes. The infiltration levels of 28 immune cells in the expression profile and their relationship to hub gene markers were analysed using single-sample GSEA (ssGSEA). In addition, the accuracy of the hub markers in diagnosing ANCA-GN was subsequently evaluated using the receiver operating characteristic curve (ROC).

**Results:**

A total of 651 differential genes were screened. Twelve co-expression modules were obtained *via* WGCNA; of which, one hub module (black module) had the highest correlation with ANCA-GN. A total of 66 intersecting genes were acquired by combining differential genes. Five hub genes were subsequently obtained by lasso analysis as potential biomarkers for ANCA-GN. The immune infiltration results revealed the most significant relationship among monocytes, CD4^+^ T cells and CD8^+^ T cells. ROC curve analysis demonstrated a prime diagnostic value of the five hub genes. According to the functional enrichment analysis of the differential genes, hub genes were mainly enhanced in immune- and inflammation-related pathways.

**Conclusion:**

B cells and monocytes were closely associated with the pathogenesis of ANCA-GN. Hub genes (*CYP3A5*, *SLC12A3*, *BGN*, *TAPBP* and *TMEM184B*) may be involved in the progression of ANCA-GN through immune-related signal pathways.

## Introduction

Antineutrophil cytoplasmic antibody (ANCA)-associated vasculitis (AAV) is an autoimmune disease characterised by ANCA-positive condition in blood circulation and inflammation and damage in small- and medium-sized blood vessels. The basic pathological characteristic of this disease is necrotising small-vessel vasculitis, with or without granuloma formation (1, 2). The clinical symptoms of AAV are complex, mainly presenting as systemic symptoms and damage to various organ systems, with the kidneys and lungs being most frequently involved. Kidney injury caused by AAV is known as ANCA-associated glomerulonephritis (ANCA-GN). ANCA-GN often progresses gradually; however, it may be acute, with short-lived and rapidly progressive GN and pulmonary haemorrhage, leading to renal and respiratory failure and even death (3–6). Moreover, there has been no consensus on the pathogenesis of AAV for a long time. Although the kidney is one of the most susceptible organs to AAV, genetic susceptibility plays a crucial role in its pathogenesis (6). Therefore, a deeper understanding of the molecular mechanisms associated with the occurrence and progression of AAV-related kidney damage is of great significance for early diagnosis and treatment of the disease and the identification of new therapeutic targets.

Despite the controversial pathogenesis of AAV, accumulating clinical and experimental evidence reveals the importance of immune cells and immune-related pathways in the progression of AAV. For example, abnormalities in both the function and count of regulatory T cells (Tregs) lead to suppression of the proliferation of autoreactive T cells, mediating the progression of AAV (7). Low expression levels of cluster of differentiation (CD)4, CD25 and CD122 (interleukin [IL]-2 receptor beta chain, a critical signal transduction molecule for T cells) on Tregs are associated with systemic vasculitis accompanied by renal involvement and its recurrence (2, 8, 9). In addition, helper and cytotoxic T cells recognise myeloperoxidase (MPO) as a vascular antigen, thereby inducing the activation of autoreactive B cells, ANCA production and leukocyte recruitment to destroy the vasculature. Depletion of CD4^+^ and CD8^+^ T cells or their cytokines can further mitigate the damage caused by anti-myeloperoxidase in GN (10, 11). These molecular and cellular experiments play the key role of immune cells and immune-related pathways in the progression of AAV; however, the molecular mechanisms determining the pathogenesis of ANCA-GN remain unclear.

As common methods in bioinformatic analysis, weighted gene co-expression network analysis (WGCNA) and most minor absolute shrinkage and selection operator (LASSO) algorithm have a wide range of applications. Specifically, WGCNA works by clustering similarly expressed genes into a single module based on the clustering principle. Compared with simple clustering analysis, this method has biological significance and can effectively achieve preliminary screening of target feature-related genes (12, 13). As a regression method, LASSO aims to clarify the specific degree of association between two related variables that have a dimensionally reduced effect compared with the traditional Cox regression and logistic regression. LASSO analysis on the genes of WGCNA can improve the accuracy of screening target feature-related genes (14). Based on the screening of differentially expressed genes (DEGs), WGCNA combined with LASSO was used to screen for key biomarkers of ANCA-GN, followed by Gene Ontology (GO), Kyoto Encyclopedia of Genes and Genomes (KEGG) and Gene Set Enrichment Analysis (GSEA) analyses of DEGs to determine their immune-related signal pathways. To the best of our knowledge, single-sample GSEA (ssGSEA) was used for the first time in this study to analyse infiltration of 28 immune cells, to gain a deeper insight into the pathogenesis of ANCA-GN and provide a new basis for further assessment of its pathogenesis and therapeutic targets.

## Materials and Methods

### Data Download

Microarray expression data for ANCA-GN and its clinical information were downloaded from the GEO (Gene Expression Omnibus, http://www.ncbi.nlm.nih.gov/geo/) database (GSE108113 and GSE104948). A total of 21 samples were included from the GSE108113 dataset, with 15 cases of ANCA-GN and six healthy controls. The sequencing platform used was GPL19983; 40 samples were included from the GSE104948 dataset, with 22 cases of ANCA-GN and 18 healthy controls.

### Identification of DEGs

Data normalisation and probe annotation were performed on the data of the GSE108113 dataset using the ‘limma’ and ‘GEOquery’ packages of R software (version 4.0.1) with DEG screening criteria of adjusted *P-value* < 0.05 and |log fold change (FC)| > 1 (15, 16).

### Construction of Gene Co-Expression Network

A weighted co-expression network was constructed for the expression profile data of the GSE108113 dataset with the help of the WGCNA package of R software, followed by a selection of genes with the top 25% absolute deviation from the median for analysis (12). The integrity of the data was checked with the ‘goodSampleGenes’ function. An ideal soft threshold (*β*) was selected and verified with the help of the ‘pickSoftThreshold’ function. The matrix data were then transformed into an adjacency matrix, followed by clustering, to identify modules based on the topological overlap. After completing the calculation of module eigengene (ME) and merging similar modules in the clustering tree according to ME, a hierarchical clustering dendrogram was drawn. Modules were combined with phenotypic data to calculate gene significance (GS) and module significance (MS) to measure the significance of genes and clinical information and analyse the correlation between modules and models. Moreover, the module membership (MM) was calculated for each gene to analyse the GS in the module.

### Screening and Validation of Hub Genes

Genes with the highest inter-module connectivity were selected as candidate hub genes. Genes with biological significance usually have higher absolute GS values. Candidate hub genes were screened against the criteria (absolute value of GS > 0.20; absolute value of MM > 0.80). The candidate hub genes were then intersected with DEGs using the ‘glmnet’ package of R software to perform LASSO analysis to screen for the final hub genes (13). Hub gene expression levels in ANCA-GN and healthy individuals were assessed with the help of box plots. Receiver operating characteristic curves (ROCs) were plotted using the MedCalc software (version 19.1) to assess the levels of hub genes distinguishing between ANCA-GN and healthy individuals. Furthermore, the expression levels and diagnostic value of the hub genes were validated with a separate external dataset (GSE104948). Meanwhile, the expression levels and diagnostic value of the hub genes in healthy individuals compared to different renal pathologies [including diabetic nephropathy (DN), focal segmental glomerular sclerosis (FSGS), and minimal change disease (MCD)] were validated using a GEO dataset (GSE104948).

### Assessment of Immune Cell Infiltration and Its Correlation With Hub Genes

The relative infiltration levels of 28 immune cells in the GSE108113 dataset were quantified using the ssGSEA algorithm (17). Violin plots were drawn to demonstrate the differential expression levels of the 28 immune infiltrating cells. Spearman correlations were calculated for 28 immune infiltrating cells with hub genes, followed by visualisation using the ‘ggplot2’ package.

### Functional Enrichment Analysis

GO analysis, KEGG analysis and GSEA of DEGs were performed using the ‘clusterProfiler’ and ‘enrichplot’ packages of R, with *P* < 0.05 being a statistically significant difference (18). Immunologic signature gene sets from the Molecular Signatures Database (MsigDB) database were used as a reference for GSEA, where gene sets with *P* < 0.05 and false discovery rate (FDR) *q-value* < 0.05 were considered significantly enriched.

## Results

### Co-Expression Network Construction and Hub Module Identification

**Figure 1:**
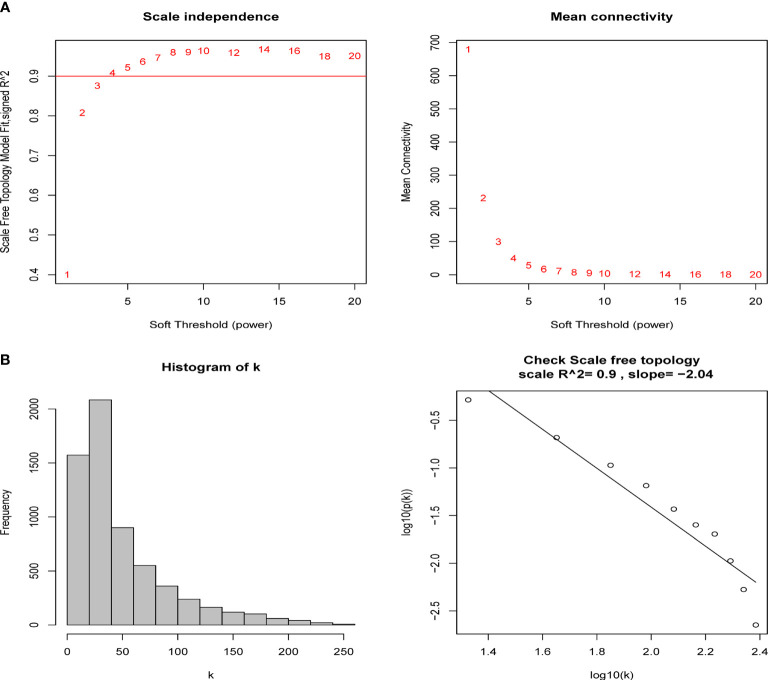
Determination of soft-thresholding power in the weighted gene co-expression network analysis (WGCNA). **(A)** Analysis of the scale-free fit index and the mean connectivity for various soft-thresholding powers (*β*). The red line indicates where the correlation coefficient is 0.9, and the corresponding soft-thresholding power is 4. **(B)** Histogram of connectivity distribution and checking the scale-free topology when *β* = 5.

**Figure 2:**
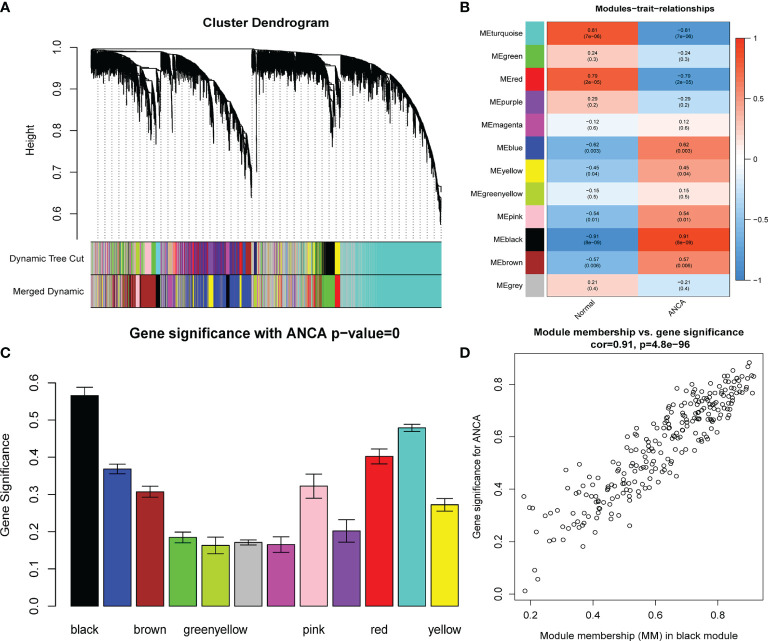
Construction of WGCNA modules. **(A)** The cluster dendrogram of the genes with median absolute deviation in the top 25%. Each branch in the figure represents one gene, and every color below represents one co-expression module. **(B)** Heatmap of the module-trait relationships. The black module was significantly associated with ANCA. **(C)** Distribution of average gene significance in the modules associated with the ANCA. **(D)** Scatter plot for correlation between gene module membership in the black module and gene significance. WGCNA, weighted gene co-expression network analysis; ANCA, antineutrophil cytoplasmic antibody.

A total of 6224 genes in the top 25% absolute deviation from the median were selected for WGCNA construction, followed by clustering of samples for processing of missing value and culling of an outlier. A soft threshold of *β* = 4 (scale-free *R^2^* = 0.9; slope = −2.04) was selected for consistency with the scale-free network (**Figure 1**). A one-step method was used to construct the co-expression matrix, and dynamic hybrid shearing was used to obtain 12 gene modules (**Figure 2A**). The correlations of the above-mentioned modules with ANCA-GN and healthy controls were presented with the help of heat maps, with one hub module (black module, including 248 genes) demonstrating the highest correlation (cor) with ANCA-GN (cor = 0.91; *P* = 8e−9) (**Figures 2B, C**). In addition, there was a good correlation between GS and MM within the black module (cor = 0.91; *P* = 4.8e−96) (**Figure 2D**). In addition, the black module was used as a hub module for subsequent analysis.

### Identification of DEGs and Screening of Hub Genes

**Figure 3:**
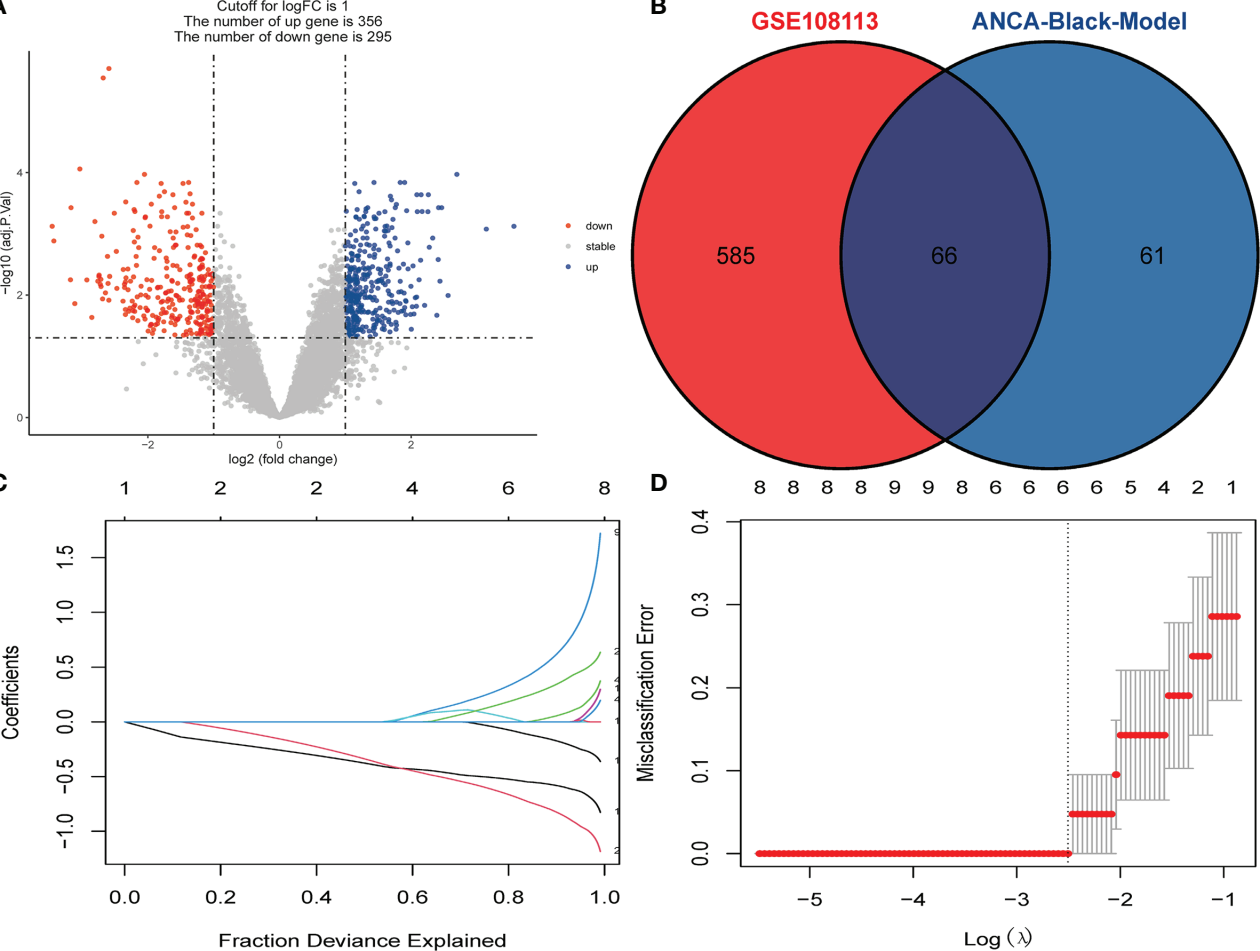
Identification of DEGs and Screening of Hub Genes. **(A)** Volcano plot for DEGs between healthy controls and ANCA-GN tissues. **(B)** Venn diagram for intersections between DEGs and the black module. **(C)** Partial likelihood deviance with changing of log (*λ*) plotted by LASSO regression in 10-fold cross-validations. Dotted vertical lines were drawn at the optimal values using the minimum criteria (lambda.min) and 1 standard error of the minimum criteria (1-SE criteria). **(D)** The LASSO coefficient profiles for five hub genes in the 10-fold cross-validation. DEGs, differentially expressed genes; ANCA-GN, Antineutrophil cytoplasmic antibody-associated glomerulonephritis; LASSO, least absolute shrinkage and selection operator.

A total of 651 DEGs, including 356 up-regulated genes and 295 down-regulated genes, were obtained based on adjusted *P-value* < 0.05 and |logFC| > 1. The DEGs were demonstrated on volcano plots (**Figure 3A**). A total of 127 genes with the highest connectivity in the black module were identified as candidate hub genes based on the screening criteria (absolute value of GS > 0.20; the absolute value of MM > 0.80). Subsequently, 66 intersecting genes were obtained based on the intersections of DEGs (**Figure 3B**). Subsequently, LASSO analysis was performed, resulting in five hub genes as follows: *CYP3A5*, *SLC12A3*, biglycan [*BGN*], *TAPBP* and *TMEM184B* (**Figures 3C, D**).

### Functional Enrichment Analysis of DEGs

**Figure 4:**
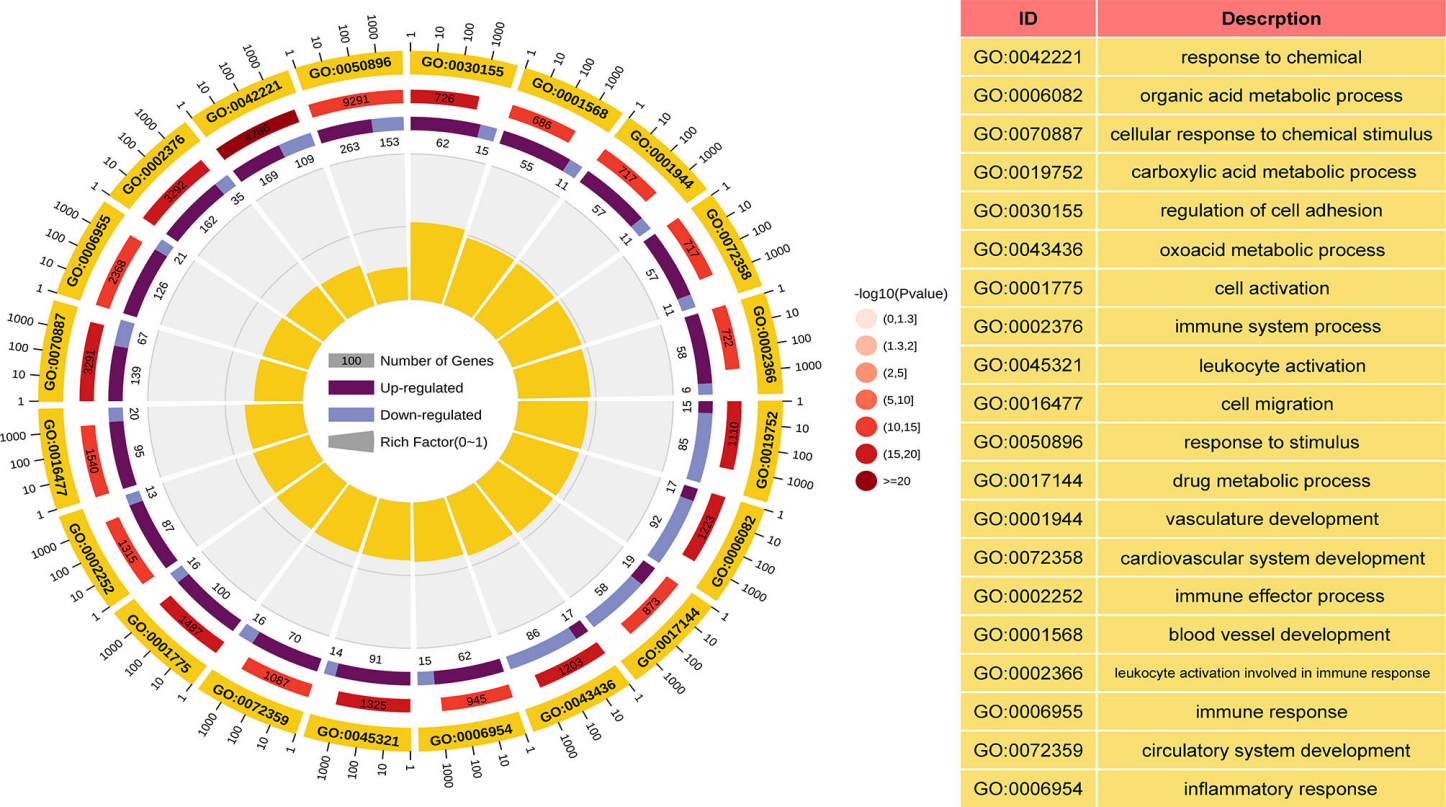
GO term analysis of DEGs in biological processes. The first lap indicates top 20 GO terms and the number of the genes corresponds to the outer lap. The second lap indicates the number of the genes in the genome background and *P* values for enrichment of the DEGs for specified biological process. The third lap indicates the ratio of the upregulated genes (deep purple) and downregulated genes (light purple). The fourth lap indicates the enrichment factor of each GO term. GO, gene oncology; DEGs, differentially expressed genes.

**Figure 5:**
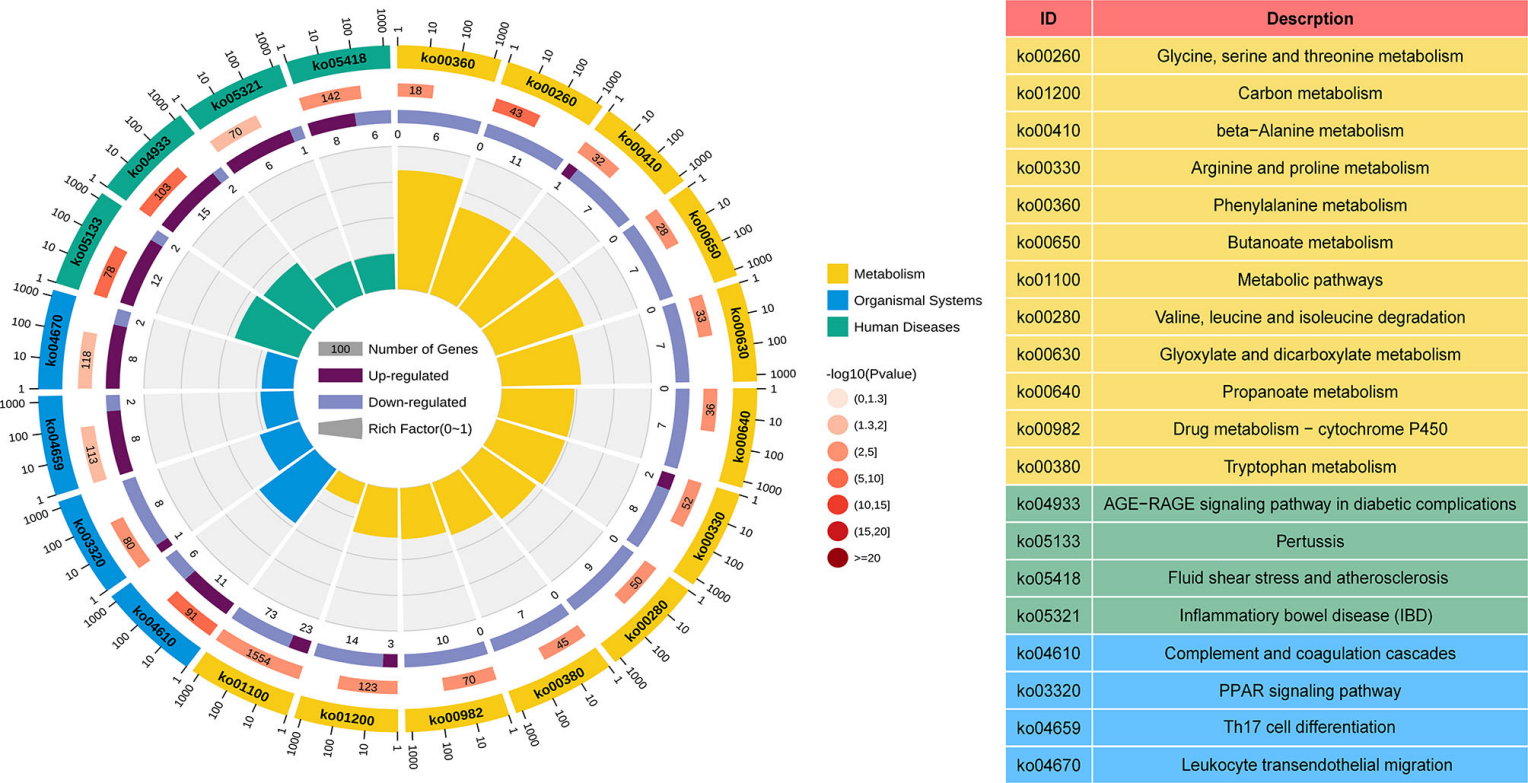
KEGG term analysis of DEGs in the metabolism processes, organismal systems, and human diseases. The first lap indicates top 20 KEGG terms and the number of the genes corresponds to the outer lap. The second lap indicates the number of the genes in the genome background and *P* values for enrichment of the DEGs. The third lap indicates the ratio of the upregulated genes (deep purple) and downregulated genes (light purple). The fourth lap indicates the enrichment factor of each KEGG term. KEGG, Kyoto Encyclopedia of Genes and Genomes; DEGs, differentially expressed genes.

GO and KEGG analyses were performed to understand the biological functions and signal pathways of DEGs associated with ANCA-GN. In terms of biological processes, GO enrichment analysis revealed that DEGs were mainly enriched in defence processes (e.g., regulation of cell adhesion, leukocyte activation, cell activation and cell migration), immune and inflammatory-related processes (e.g., immune response, inflammatory response and immune effector process) and vascular development (e.g., vasculature development, cardiovascular system development, blood vessel development and circulatory system development) (**Figure 4**). Analysis of the KEGG signal pathways revealed that DEGs were mainly enriched in pathways associated with immune- and inflammatory-related diseases (e.g., advanced glycation end product-receptor for advanced glycation end product (AGE-RAGE) signalling pathway in diabetic complications, pertussis, fluid shear stress, atherosclerosis and inflammatory bowel disease) and immune-related pathways (e.g., peroxisome proliferator-activated receptors (PPAR) signalling pathway, type 17 T helper cells (Th17) cell differentiation and leukocyte transendothelial migration) (**Figure 5**). In conclusion, these results identified the biological processes and aberrant signal pathways involved in the progression of ANCA-GN.

### Identification of Hub Gene Expression Levels and Diagnostic Value

**Figure 6:**
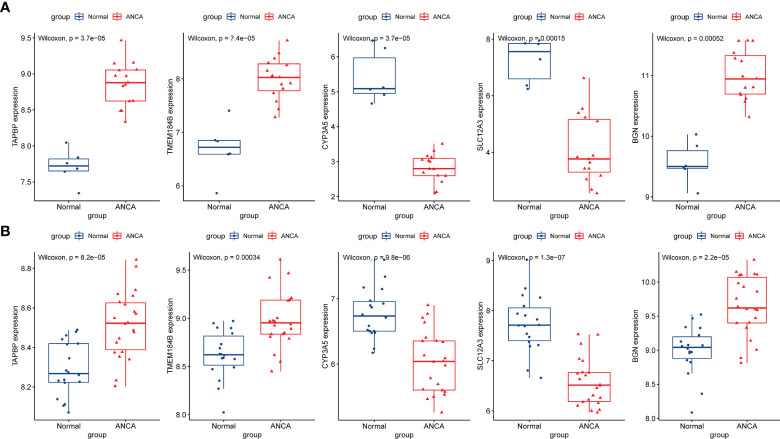
Validation of hub genes in the gene expression level. **(A)** Validation of hub genes in the GSE108113. *TAPBP*, *TMEM184B*, and *BGN* were significantly higher expression in ANCA-GN compared with healthy controls, while *CYP3A5* and *SLC12A3* were significantly lower expression in ANCA-GN tissues compared with healthy controls. **(B)** Validation of hub genes in the GSE104948 and the results were the same as the results of the GSE108113. ANCA-GN, Antineutrophil cytoplasmic antibody-associated glomerulonephritis.

**Figure 7:**
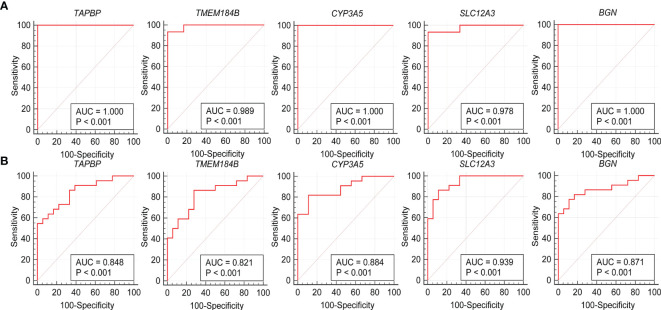
Validation of hub genes in the diagnostic value. **(A)** Validation of hub genes in the GSE108113. ROC curves and AUC statistics are used to evaluate the capacity to discriminate ANCA-GN from healthy controls with excellent sensitivity and specificity. **(B)** Validation of hub genes in the GSE104948 and the results were the similar as the results of the GSE108113. These findings indicated these five hub genes have excellent diagnostic efficiency in ANCA-GN. ANCA-GN, Antineutrophil cytoplasmic antibody-associated glomerulonephritis; ROC, receiver operating characteristic; AUC, area under the curve.

The expression levels of the five hub genes were validated using box plots. **Figure 6A** demonstrates significantly higher expression levels of *TAPBP* (*P* = 3.7e−05), *TMEM184B* (*P* = 7.4e−05) and *BGN* (*P* < 0.001) in ANCA-GN tissues than in healthy controls and significantly lower expression levels of *CYP3A5* (*P* = 3.7e−05) and *SLC12A3* (*P* < 0.001) in ANCA-GN tissues than in healthy controls. Subsequently, the expression levels of these five hub genes were further validated in a separate external dataset, GSE104948 (**Figure 6B**). Where the expression levels of these hub genes in DN, FSGS and MCD were also validated. The expression levels of *TMEM184B* (*P* = 0.0023) and *BGN* (*P* = 0.001) were significantly higher in DN tissues than in healthy controls, while *SLC12A3* (*P* = 0.014) was significantly lower in DN tissues than in healthy controls (**Supplementary Figure S1A**); the expression levels of *TAPBP* (*P* = 0.0035), *TMEM184B* (*P* < 0.001) and *BGN* (*P* = 0.025) were significantly higher in FSGS tissues than in healthy controls, while *CYP3A5* (*P* = 0.0012) and *SLC12A3* (*P* < 0.001) was significantly lower in FSGS tissues than in healthy controls (**Supplementary Figure S1B**); the expression levels of *TMEM184B* (*P* = 0.019) was significantly higher in MCD tissues than in healthy controls, while *CYP3A5* (*P* = 0.007) and *SLC12A3* (*P* < 0.001) in MCD tissues were significantly lower than those of healthy controls (**Supplementary Figure S1C**). For ROC curve analysis, the area under the curve (AUC) values of the five hub genes were compared to assess their sensitivity and specificity for the diagnosis of ANCA-GN. The AUC values > 0.95 in all five hub genes indicated the high diagnostic value of these genes for ANCA-GN (**Figure 7A**). To confirm their clinical utility, the diagnostic value of the above-mentioned five hub genes was further validated in the GSE104948 dataset. Four hub genes had AUC values > 0.85, whereas the *TMEM184B* gene had an AUC value of 0.821 (**Figure 7B**). The results in this dataset for DN, FSGS and MCD showed that three of the five hub genes were diagnostically significant for DN with a maximum AUC value of 0.905 (*BGN*), the maximum AUC value in FSGS was 0.877 (*TMEM184B*), and the maximum AUC value in MCD was 0.869 (*SLC12A3*), shown in **Supplementary Figure S2**.

### Immune Cell Infiltration and Its Correlation With Hub Genes

**Figure 8:**
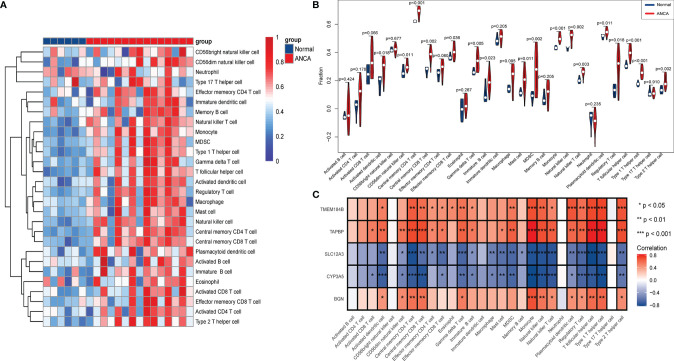
Analysis of immune landscape associated with ANCA-GN. Heatmap **(A)** and violin plot **(B)** showing the distribution of 28 types of immune cells in healthy control and ANCA-GN tissues. **(C)** The relationship between five hub genes and immune cell infiltration. ANCA-GN, Antineutrophil cytoplasmic antibody-associated glomerulonephritis.

To further investigate the differences in immune cell infiltration between ANCA-GN and healthy controls, their relationship was assessed using the ssGSEA algorithm. The distribution of 28 immune cells in the GSE108113 sample is demonstrated in **Figure 8A**. The results of the immune cell infiltration analysis demonstrated a significantly higher infiltration of monocytes, CD4^+^ T cells, CD8^+^ T cells, Tregs and natural killer (NK) cells in ANCA-GN than in healthy tissue, suggesting that these cells are essential in the progression of ANCA-GN (**Figure 8B**). Correlation analysis of 28 immune cells with hub genes demonstrated positive correlations of monocytes with *TMEM184B* (cor = 0.696; *P* < 0.001), *TAPBP* (cor = 0.830; *P* < 0.001) and *BGN* (cor = 0.687; P < 0.001) and negative correlations with *SLC12A3* (cor = −0.768; *P* < 0.001) and *CYP3A5* (cor = −0.827381253; *P* < 0.001). Type 1 T helper (Th1) cells were positively correlated with *TMEM184B* (cor = 0.726; *P* < 0.001), *TAPBP* (cor = 0.696; *P* < 0.001) and *BGN* (cor = 0.612; *P* = 0.003) and negatively correlated with *SLC12A3* (cor = −0.762; *P* < 0.001) and *CYP3A5* (cor = −0.792; *P* < 0.001). Tregs had a positive correlation with *TMEM184B* (cor = 0.586; *P* = 0.005), *TAPBP* (cor = 0.628; *P* = 0.002) and *BGN* (cor = 0.445; *P* = 0.043) and negative correlation with *SLC12A3* (cor = −0.670; *P* < 0.001) and *CYP3A5* (cor = −0.673; *P* < 0.001). In addition, activated dendritic cells, central memory CD4^+^ T cells, central memory CD8^+^ T cells, gammadelta T cells, NK cells, plasmacytoid dendritic cells and T follicular helper cells were positively correlated with *TMEM184B*, *TAPBP* and *BGN* (all *P* < 0.05) and negatively correlated with *SLC12A3* and *CYP3A5* (all *P* < 0.05) (**Figure 8C**). These results further demonstrated the crucial role played by these immune cells in the progression of ANCA-GN.

### Enrichment Analysis of GSEA Immune Signature Gene Sets

**Figure 9:**
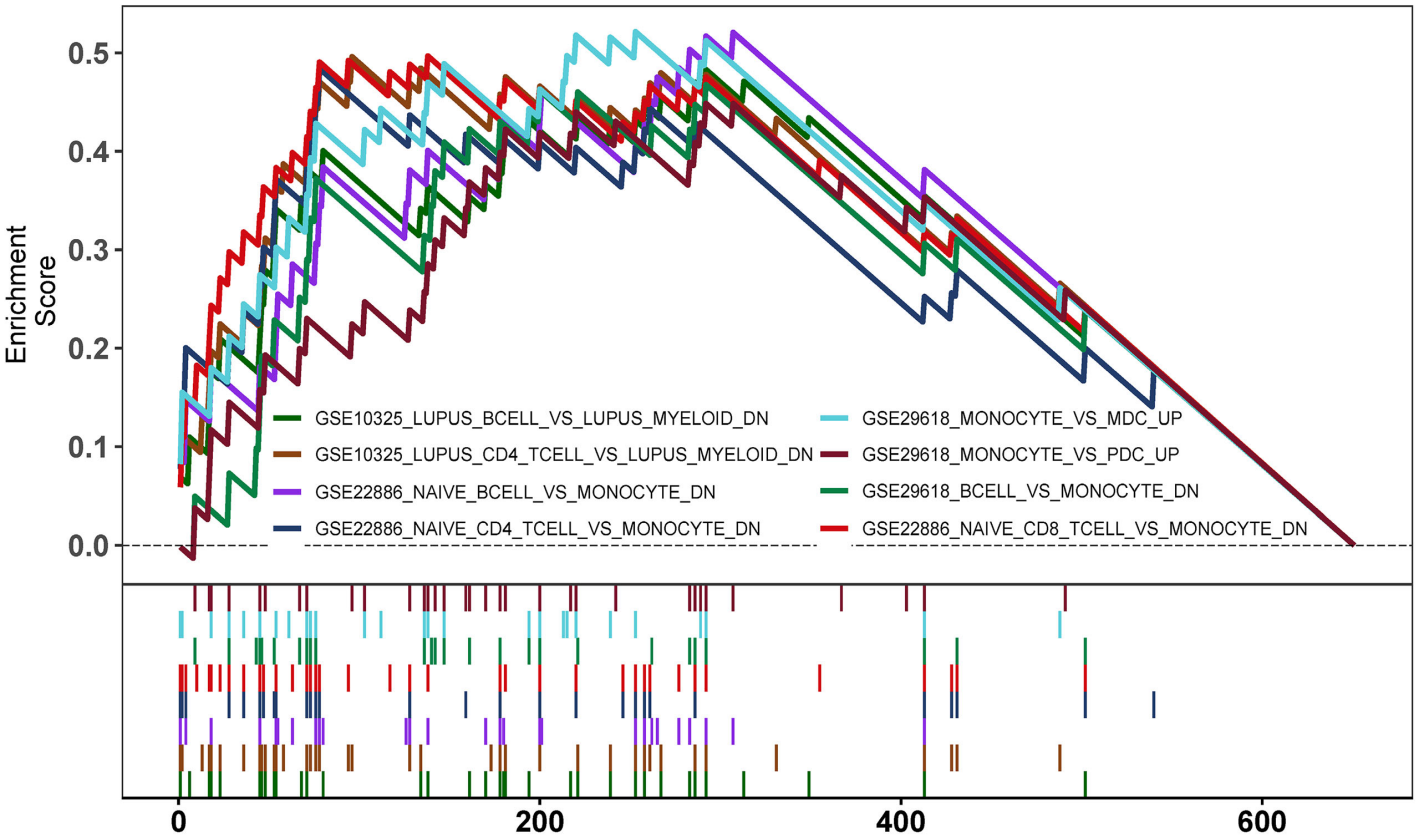
Enrichment plot for GSEA Immunologic signature database.

To explore the potential mechanisms of immune function during ANCA-GN progression, the immunologic signature gene sets from the MsigDB database were used as a reference for the GSEA of DEGs. A total of 383 gene sets were significantly enriched (|normalised enriched score (NES)| > 1; FDR *q-value* < 0.05). These gene sets were mainly enriched higher in CD4^+^ T cells, CD8^+^ T cells, B cells, monocytes, peripheral blood mononuclear cells (PBMCs) and dendritic cells (DCs). The top 15 enriched gene sets are listed in **Table 1**. These results demonstrated the crucial role played by immune-related genes in the occurrence and progression of ANCA-GN (**Figure 9**).

**Table 1:** Top 15 significant immunological signature enriched by DEGs in GSEA.

Gene set name	NES	pvalue	NOM p-val	FDR q-val
GSE22886 Naive CD8 T cell vs monocyte down	3.159	1.76E-07	4.91E-05	3.51E-05
GSE10325 Lupus CD4 T cell vs lupus myeloid down	3.173	2.11E-07	4.91E-05	3.51E-05
GSE10325 Lupus B cell vs lupus myeloid down	2.950	1.42E-06	< 0.001	< 0.001
GSE29618 Monocyte vs MDC up	2.891	2.85E-06	< 0.001	< 0.001
GSE22886 Naïve B cell vs monocyte down	2.888	2.94E-06	< 0.001	< 0.001
GSE21670 STAT3 ko vs WT CD4 T cell TGF-β IL6 treated down	2.894	8.42E-06	< 0.001	< 0.001
GSE36009 WT vs NLRP10 ko DC down	2.672	1.51E-05	< 0.001	< 0.001
GSE29618 Monocyte vs PDC up	2.720	2.74E-05	0.0019	< 0.001
GSE24142 Early thymic progenitor vs DN2 thymocyte fetal up	2.763	2.78E-05	0.001	< 0.001
GSE22886 Naïve CD4 T cell vs monocyte down	2.760	3.83E-05	0.002	0.001
GSE23568 Ctrl transduced vs WT CD8 T cell down	2.606	4.79E-05	0.002	0.001
GSE40274 Ctrl vs IRF4 transduced activated CD4 T cell up	2.423	< 0.001	0.004	0.003
GSE29618 B cell vs monocyte down	2.588	< 0.001	0.004	0.003
GSE26890 CXCR1 neg vs pos effector CD8 T cell up	2.404	< 0.001	0.004	0.003
GSE28783 Ctrl anti-mir vs untreated atherosclerosis macrophage up	-2.195	< 0.001	0.009	0.006

DEGs, differentially expressed genes; GSEA, gene set enrichment analysis; NES, normalized enrichment score; NOM, nominal; FDR, false discovery rate.

## Discussion

The application of high-throughput microarray technology has led to a more rapid and efficient bioinformatic approach to screen for core genes associated with disease occurrence and progression mechanisms, providing a basis for disease diagnosis, treatment and new drug design. In this study, DEGs were discovered to be mainly enriched in leukocyte activation, immune response, inflammatory response, immune effector process and vascular development, which are associated with the pathogenesis of ANCA-GN (2, 19). Previous studies have identified a facilitative effect of high expression levels of inflammatory cytokines (IL-1, tumour necrosis factor-alpha [TNF-α] and IL-6), cell adhesion molecules and cell chemokines on immune cell recruitment, leading to disruption of the vascular system (10, 20, 21). Analysis of the KEGG signal pathway revealed that DEGs were mainly enriched in pathways associated with immune- and inflammatory-related diseases, immune-related pathways (PPAR signalling pathway, Th17 cell differentiation and leukocyte transendothelial migration). The effector molecules of Th17 cells primarily include the cytokine IL-17. Several physiological effects mediated by Th17 are produced primarily through IL-17 (22, 23). IL-17 acts on various cell types and induces the production of many cytokines, including TNF, granulocyte–macrophage colony-stimulating factor (GM-CSF), IL-6, IL-1β and several chemokines (24–26). In addition, Th17 cells secrete several other cytokines with significant biological effects (24). Previous studies have found elevated levels of Th17 and IL-17 in the peripheral blood of patients with AAV (27). Ifuku et al. found increased expression levels of Th17-related cytokines such as IL-17 and IL-6 in patients with AAV (28). IL-17 promotes the secretion of pro-inflammatory cytokines such as TNF-α and IL-1β and induces CXC chemokine release, upregulates endothelial cell adhesion molecules and activates and recruits neutrophils (20, 29). Knockdown of IL-17A can reduce neutrophil recruitment, block inflammatory cell infiltration and inhibit delayed-type hypersensitivity. In other words, anti-IL-17 treatment can reduce symptoms in patients (30). Velden et al. detected a large number of polymorphonuclear neutrophils expressing IL-17 in the glomerular and tubular mesenchyme of the kidney. During the acute phase of the disease, neutrophils acted as an important early intrinsic source of IL-17, mediating persistent renal inflammatory injury. Serum creatinine levels were positively correlated with renal tubulointerstitial IL-17^+^ neutrophils and IL-17^+^ T cells (31), suggesting a critical role for balanced differentiation of Th cells for immune and host protection (26). The better agreement of these studies with the results of DEGs enrichment suggested the presence of genes in DEGs that play a key role in the occurrence and progression of ANCA-GN.

WGCNA, which has been widely used in recent years, was used to avoid the disadvantages of traditional DEG-based screening methods that are only used to study the dataset locally, which is very likely to miss the core molecules in the regulatory process and make it difficult to explore the biological system as a whole. Systematic mapping of individual biological interaction networks can identify the core molecules associated with prognosis (32, 33). Based on the genes with high correlation with ANCA-GN mined by WGCNA, the results obtained in this study were intersected with the previous DEGs to obtain intersecting genes with both difference and correlation. Subsequently, the following five hub genes were eventually obtained *via* LASSO analysis: *CYP3A5*, *SLC12A3*, *BGN*, *TAPBP* and *TMEM184B*. The expression levels of the five hub genes differed significantly in healthy and ANCA-GN tissues. Specifically, *TAPBP*, *TMEM184B* and *BGN* were all significantly highly expressed in ANCA-GN tissues, whereas *CYP3A5* and *SLC12A3* demonstrated significantly lower expression levels.

Transporter associated with antigen processing (TAP)-binding protein (TAPBP), encoding tapasin involved in class I antigen processing, is an important component of the major histocompatibility complex (MHC)-I signalling pathway (34, 35). The antigen-promoting properties of TAPBP identified in several studies correlated with CD8^+^ cytotoxic T lymphocyte infiltration, mediating inflammatory injury (34, 36). *BGN* is a member of the small leucine-rich proteoglycans (SLRPs) family and is widely distributed in various extracellular matrices (ECM) tissues such as cartilage, tendons and fibrous tissue and is an important component of the ECM (37). In the presence of tissue stress or injury, *BGN* functions as damage-associated molecular patterns (DAMPs) after hydrolytic release from the ECM (38, 39). The intact *BGN* in macrophages and dendritic cells acts as an endogenous ligand for toll-like receptor 2 (TLR2) and TLR4, resulting in rapid activation of the p38 mitogen-activated protein kinase (p38 MAPK) and nuclear factor-kappa B (NF-κB) pathway. The activation of these signal pathways leads to the secretion of cytokines such as TNF-α and IL-1β, followed by the recruitment of more inflammatory cytokines to the infection sites by chemokines (40–44). Another study suggested that down-regulation of *BGN* levels in models such as unilateral ureteral obstruction and lupus nephritis effectively reduces the expression levels of nucleotide-binding oligomerisation domain-like receptor protein 3 (NLRP3) and caspase-1 activity. In this way, the number of renal TNF-α, IL-1β and infiltrating monocytes is significantly reduced, thus providing renal protection (40, 45–48). The cytochrome P450 (CYP) family 3 subfamily A member 5 (*CYP3A5*), an important CYP450 enzyme family, is found mainly not only in the liver and intestine but also in the prostate and kidney. A review by Woolbright BL et al. elucidated the effects on the expression levels and enzymatic activity of CYPs in the liver in the context of inflammation and infection in the organisms (49). Several studies have identified the regulatory role of inflammation caused by infections in bacteria, viruses and parasites and inflammatory diseases such as rheumatoid arthritis in the expression of CYPs (50–52). CYPs are largely under-expressed in the inflammatory disease setting (53). The above-mentioned studies suggest that *TAPBP*, *BGN* and *CYP3A5* are closely associated with AAV-induced renal injury. Currently, the relationship between *TMEM184B* and *SLC12A3* and the progression of AAV remains unclear and needs to be further explored. In the meantime, we also compared the expression levels of the above genes in healthy tissues with DN, FSGS and MCD tissues, also with some degree of difference. However, the diagnostic efficiency of the above genes in these diseases was inferior to that of ANCA-GN, suggesting on this hand that the above hub genes may also be involved in renal diseases due to other causes, but with a more vital specificity than ANCA-GN, while on the other hand, potentially implicating that there are some links between immune-related glomerular diseases, through selected genes (54).

The above-mentioned studies confirm the close association of DEGs with immune response, inflammatory response, Th17 cell differentiation and chemokines. Many cytokines and chemokines produced by the interaction of T cells, B cells, macrophages, monocytes and other cells are closely associated with AAV progression (26, 39, 55–57). To the best of our knowledge, the ssGSEA algorithm was used for the first time in this study to analyse the infiltration of 28 immune cell types in ANCA-GN tissue. Compared with healthy tissue, ANCA-GN tissue had significantly higher numbers of CD4^+^ T cells, CD8^+^ T cells, Th cells, monocytes and DCs. Th17 cells are derived from CD4^+^ T cells. Previous studies have, however, suggested that CD4^+^ T cells can only differentiate into Th1 and Th2 cells. Th17 cells were first proposed in 2005 and were named for their ability to secrete high levels of IL-17 (23) specifically. Studies have demonstrated that sustained activation of T cells, particularly abnormal activation of Th17 cells is an essential mechanism in the autoimmune pathogenesis of AAV (27). Treg, a subset of CD4^+^ T cells that can balance Th cells and highly express CD25 and the unique transcription factor forkhead box P3 (FOXP3), has been demonstrated to play a crucial role in the maintenance of immune stability and occurrence and progression of autoimmune diseases (58, 59). In addition to their involvement in the pathogenesis of AAV as precursors to ANCA-producing plasma cells, B cells can present antigens to T cells to stimulate T cell activation and secrete pro-inflammatory response factors (e.g., IL-6 and TNF) that decrease the anti-inflammatory activity of Tregs and increase the differentiation of effector T cells (55, 58, 60). These results demonstrated the crucial role played by disturbances in T-cell immune homeostasis in the progression of AAV (27, 57, 59). Moreover, it has been demonstrated that monocytes and macrophages play a crucial role in the advancement of AAV (61). The immune infiltration analysis in this study revealed significantly higher levels of CD4^+^ T cells, monocytes and Th cells in the tissues of patients with ANCA-GN than in healthy individuals. To explore the potential mechanisms of immune function during the progression of ANCA-GN, the immunologic signature gene sets from the MsigDB database were used as a reference for the GSEA of DEGs. The results revealed that DEGs were mainly enhanced in CD4^+^ T cells, CD8^+^ T cells, B cells, monocytes, PBMCs and DCs, suggesting that the occurrence and progression of ANCA-GN are mediated by the abnormal activation of T cells and B cells and several cytokines and chemokines produced by their interaction.

In conclusion, with the help of WGCNA and LASSO combined with bioinformatic analysis using ssGSEA, one hub module (black module) and five hub genes (*CYP3A5*, *SLC12A3*, *BGN*, *TAPBP* and *TMEM184B*) that might be involved in the progression of ANCA-GN were screened. This study provided preliminary insights into the immune infiltration pattern of ANCA-GN and its potential immune regulatory mechanisms. In terms of follow-up studies, prospective and large-sample studies will be used to further screen for diagnostic markers with high sensitivity and specificity for ANCA-GN to reduce invasive testing and provide a reference for early diagnosis and targeted drug research for ANCA-GN.

## Data Availability Statement

The datasets presented in this study can be found in online repositories. The names of the repository/repositories and accession number(s) can be found in the article/**Supplementary Material**.

## Author Contributions

MX, RY, and DC developed the research question. MX and DC wrote the first draft of the manuscript. All authors contributed to the development of the review protocol, data analysis, and refining of the manuscript, and approved the final manuscript. MX and DC critically read and revised the manuscript before submission.

## Funding

This work was supported by grant from Nanchong School Science and Technology Strategic Cooperation Project (20SXQT0117) and Sichuan Traditional Chinese Medicine Research Project (2020LC0146).

## Conflict of Interest

The authors declare that the research was conducted in the absence of any commercial or financial relationships that could be construed as a potential conflict of interest.

## Publisher’s Note

All claims expressed in this article are solely those of the authors and do not necessarily represent those of their affiliated organizations, or those of the publisher, the editors and the reviewers. Any product that may be evaluated in this article, or claim that may be made by its manufacturer, is not guaranteed or endorsed by the publisher.

## Supplementary Material

Supplementary Figure 1 Validation of hub genes in the gene expression level. **(A)** Validation of hub genes in the DN samples. *TMEM184B* and *BGN* were significantly higher expression in DN than healthy controls, while *SLC12A3* was significantly lower in the expression of DN tissues than healthy controls. However, *TAPBP* was higher expression in DN than healthy controls, while *CYP3A5* was lower expression in DN, and the result revealed no significant correlation. **(B)** Validation of hub genes in the FSGS samples and the results were the same as the results of the DN. **(C)** Validation of hub genes in the MCD samples and the results were the same as the DN and FSGS. ANCA-GN, antineutrophil cytoplasmic antibody-associated glomerulonephritis; DN, diabetic nephropathy; FSGS, focal segmental glomerular sclerosis; MCD, minimal change disease.

Supplementary Figure 2 Validation of hub genes in the diagnostic value. **(A)** Validation of hub genes in the DN samples. ROC curves and AUC statistics are used to evaluate the capacity to discriminate DN from healthy controls. **(B)** Validation of hub genes in the FSGS samples and the results were the same as the results of the DN. **(C)** Validation of hub genes in the MCD samples and the results were the same as the results of the DN and FSGS. ANCA-GN, antineutrophil cytoplasmic antibody-associated glomerulonephritis; DN, diabetic nephropathy; FSGS, focal segmental glomerular sclerosis; MCD, minimal change disease; ROC, receiver operating characteristic; AUC, area under the curve.

## Abbreviations

ANCA, Antineutrophil cytoplasmic antibody; AAV, ANCA-associated vasculitis; ANCA-GN, ANCA-associated glomerulonephritis; DN, Diabetic nephropathy; FSGS, Focal segmental glomerular sclerosis; MCD, Minimal change disease; IL, Interleukin; MPO, Myeloperoxidase; WGCNA, Weighted gene co-expression network analysis; LASSO, Least absolute shrinkage and selection operator; DEG, Differentially expressed genes; GO, Gene Ontology; KEGG, Kyoto Encyclopedia of Genes and Genomes; GSEA, Gene Set Enrichment Analysis; ssGSEA, Single-sample GSEA; FC, fold change; ME, Module eigengene; GS, Gene significance; MS, Module significance; MM, Module membership; ROC, Receiver operating characteristic; PPAR, Peroxisome proliferator-activated receptors; Th17, Type 17 T helper; SLRPs, Small leucine-rich proteoglycans; ECM, Extracellular matrices; TLR2, Toll-like receptor 2; NF-κB, Nuclear factor-kappa B; NLRP3, Nucleotide-binding oligomerisation domain-like receptor protein 3; FOXP3, Forkhead box P3.
