# Supplementary figures and images for: Identification of Hub Biomarkers and Immune-Related Pathways Participating in the Progression of Antineutrophil Cytoplasmic Antibody-Associated Glomerulonephritis
Source: Front Immunol. 2022 Jan 5;12:809325. doi: 10.3389/fimmu.2021.809325 (PMC8766858; doi:10.3389/fimmu.2021.809325)

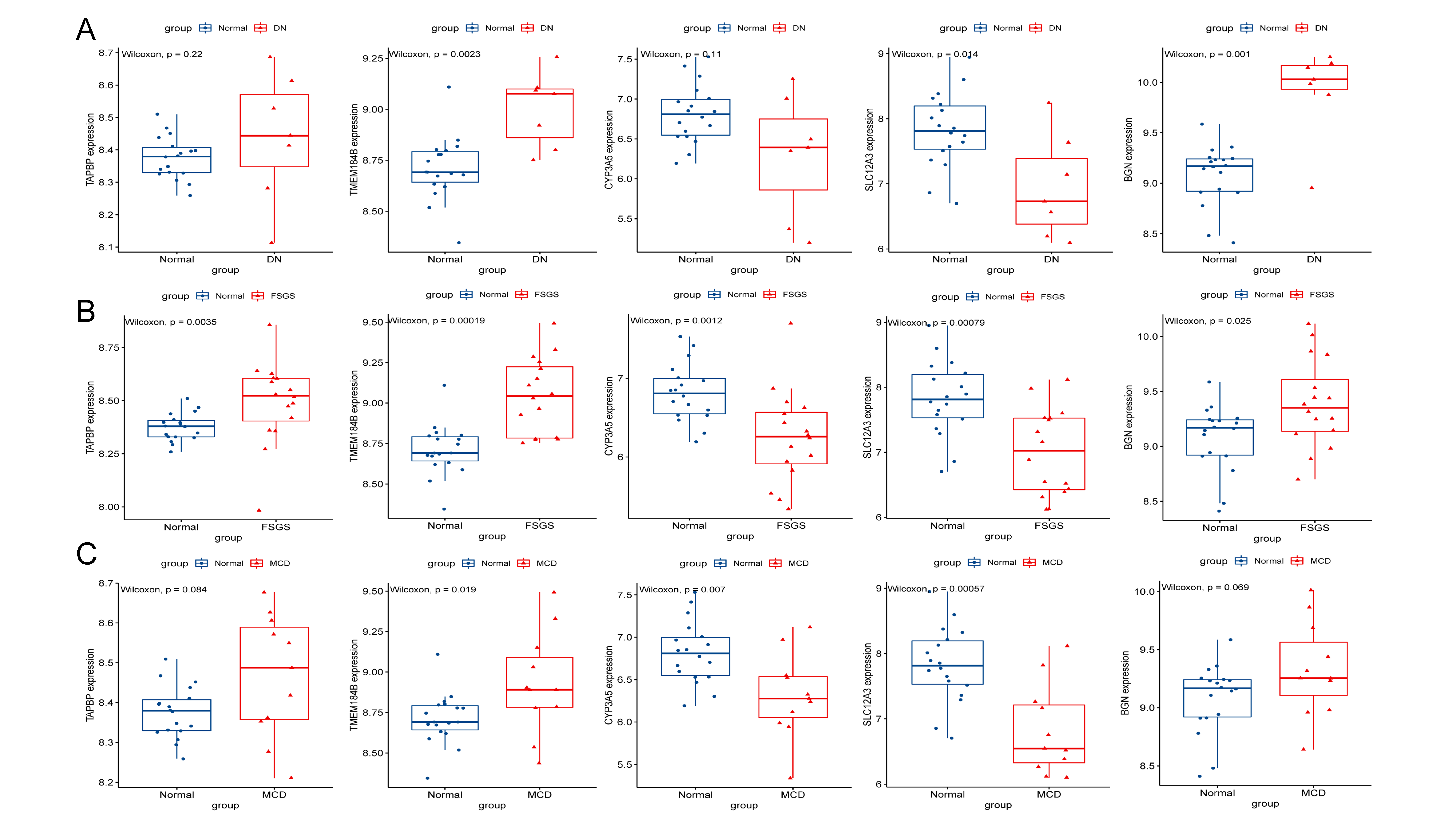

Supplement: Supplementary Figure 1 — Validation of hub genes in the gene expression level. (A) Validation of hub genes in the DN samples. TMEM184B and BGN were significantly higher expression in DN than healthy controls, while SLC12A3 was significantly lower in the expression of DN tissues than healthy controls. However, TAPBP was higher expression in DN than healthy controls, while CYP3A5 was lower expression in DN, and the result revealed no significant correlation. (B) Validation of hub genes in the FSGS samples and the results were the same as the results of the DN. (C) Validation of hub genes in the MCD samples and the results were the same as the DN and FSGS. ANCA-GN, antineutrophil cytoplasmic antibody-associated glomerulonephritis; DN, diabetic nephropathy; FSGS, focal segmental glomerular sclerosis; MCD, minimal change disease. [file Image_1.tiff]

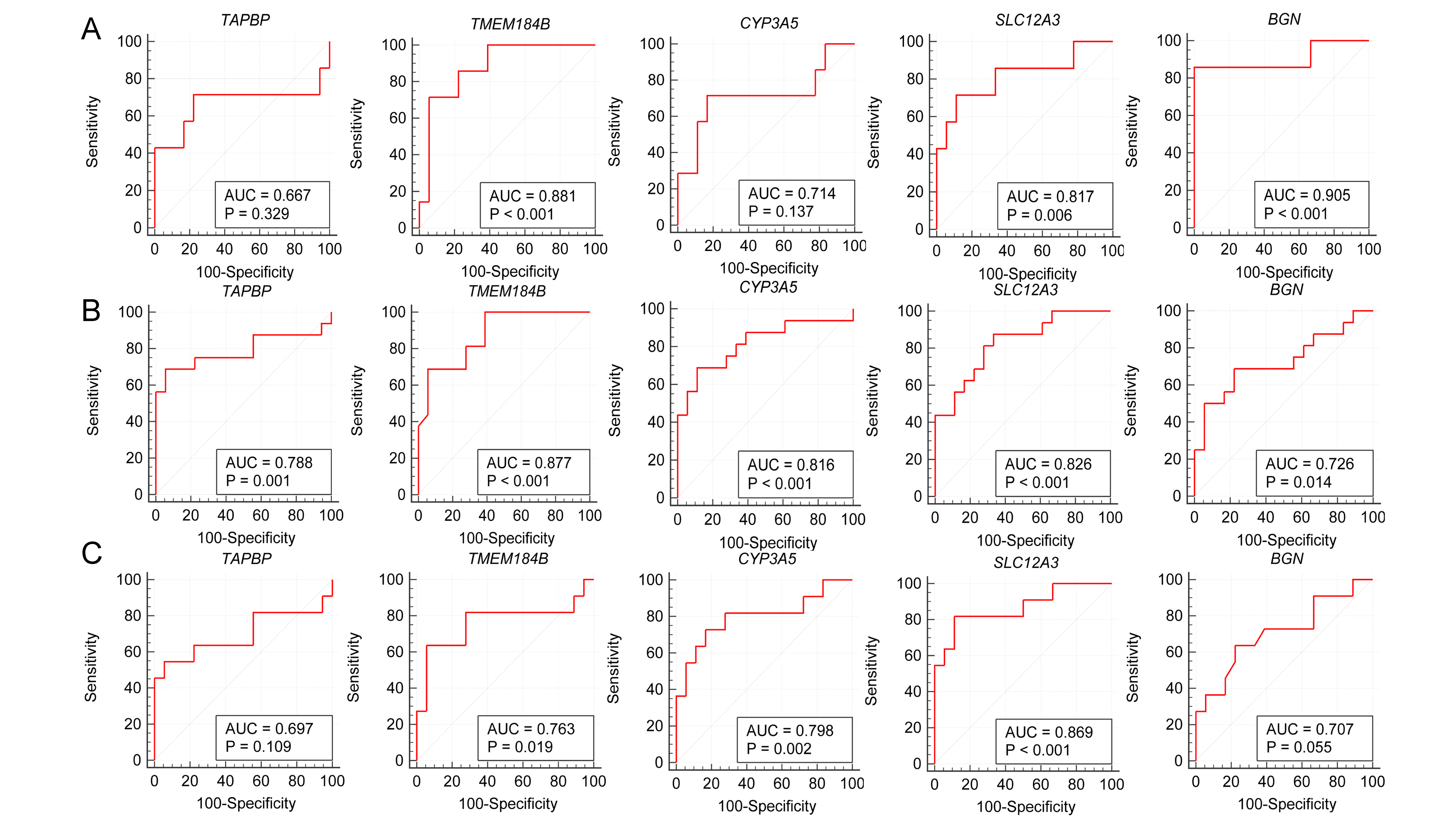

Supplement: Supplementary Figure 2 — Validation of hub genes in the diagnostic value. (A) Validation of hub genes in the DN samples. ROC curves and AUC statistics are used to evaluate the capacity to discriminate DN from healthy controls. (B) Validation of hub genes in the FSGS samples and the results were the same as the results of the DN. (C) Validation of hub genes in the MCD samples and the results were the same as the results of the DN and FSGS. ANCA-GN, antineutrophil cytoplasmic antibody-associated glomerulonephritis; DN, diabetic nephropathy; FSGS, focal segmental glomerular sclerosis; MCD, minimal change disease; ROC, receiver operating characteristic; AUC, area under the curve. [file Image_2.tiff]
